# Soil Injection Technology Using an Expandable Polyurethane Resin: A Review

**DOI:** 10.3390/polym13213666

**Published:** 2021-10-25

**Authors:** Mohanad Muayad Sabri Sabri, Nikolai Ivanovich Vatin, Kifayah Abood Mohammed Alsaffar

**Affiliations:** 1 Peter the Great St. Petersburg Polytechnic University, 195251 St. Petersburg, Russia; 2 People’s Friendship University of Russia, 117198 Moscow, Russia; vatin@mail.ru; 3 The University of Mashreq, Baghdad 10023, Iraq; kiffaya_alsaffar@yahoo.com

**Keywords:** expanding polyurethane resin, geopolymers, soil stabilization, ground improvement, foundation lifting, slab lifting, foundation remediation, building restorations, soil, sand, clay

## Abstract

The soil injection, using an expandable polyurethane resin, holds a unique potential for settlement compensation, lifting, and strengthening the foundations of existing buildings and structures. Although various research and case studies regarding this technology have been published, these studies emphasized the technology’s effectiveness in the rapid lifting process. Nevertheless, there is no complete understanding of the technology, yet, that gathers necessary data leading to a better recognition for this technology in the theoretical understanding and the practical applications. This article aims to provide a comprehensive understanding of this technology. The injection process, the resin’s mechanism, and actual propagation in the soil’s massive, the modified physic-mechanical properties of the soil, the expansion process, the consumption of the resin, and the durability are extensively reviewed in this article. Besides that, this article aims to demonstrate the advantages and limitations of this technology in practical applications. The review also explores the existing finite element models used to calculate the strength and stiffness parameters, evaluating the bearing capacity of the composite (soil-resin) and the settlement after the injection process.

## 1. Introduction

Ground modification is defined as an artificial change in the characteristics of the foundation’s soil to provide better performance under design and reconstruction when subjected to operational loading conditions. These objectives can be accomplished nowadays using a large variety of geotechnical methods and technologies that contribute to significant changes in the physic-mechanical or chemical characteristics of the foundation’s soil, leading to improvement of unsatisfactory ground conditions, mainly in the existing constructions where the replacement of the soil is not feasible for many environmental, technical, or economic reasons [1,2,3,4].

Several aspects affect the state of the foundations, including geological (natural) and other industrial (technical) factors, which, by their advent, create the necessity to obtain and use different methods and techniques, according to their availability in countries and by the type of the functions required, to solve particular foundation problems in a manner appropriate to the scope of the problems, the conditions of the foundations, and the desired results.

According to the following authors [5,6,7,8,9,10,11,12,13,14,15,16,17,18,19,20,21,22,23,24,25,26,27,28,29,30,31,32,33,34,35,36,37,38,39,40,41,42,43,44,45,46,47,48,49,50,51] and others, the main problems that cause defects and failure of the foundations, necessitating the use of ground modification methods, are classified to:

- Geological (natural) reasons: summarized in a consequence of factors associated with the native state and strength of the foundation’s soil, based on various soil features such as soil types, soil formation, organic matters, weather conditions, and other natural factors that affect the soil quality. Consequently, the foundations and infrastructures, such as roads or aerodromes built on a particular soil are affected by the advent of such soil problems. As a result, it leads to the appearance of the foundation’s imperfections threatening the whole overlaying constructions status, varying from minor defects to the collapse of the construction due to the foundation’s failure. According to the following authors [7,8,9,10,11,12,13,14,15,16,17,18,19,20,21,22,28,39,47,48,49,50,51], these factors could be sorted as Collapsible/Metastable soil, Liquefiable Soil, Expansive soil, Slope instability, seismic impact, seasonal variation of ground water level, and other natural geological and environmental reasons [52,53,54,55,56,57,58,59,60]
- Industrial (technical) reasons: summarized as complex factors caused by engineering errors, such as inadequate preliminary design, insufficient or incorrect geotechnical investigations, poor quality of construction work, violation of building codes, and poor soil compaction during the construction process. In addition, there are aspects related to changes in the capacity concerning a particular project, the poor quality of the construction materials, reconstruction processes, cities planning and developments, the insufficient distance between the adjacent foundations, vibration effect of the neighborhood construction equipment, the changes in the groundwater level due to inappropriate seepage network, and other factors. These factors are extensively studied in the following authors’ work: [6,21,23,24,25,26,27,29,30,31,32,33,34,35,36,37,38,40,41,42,43,44,45,46,49,61,62,63] and many others.

In this regard, several techniques have been developed worldwide to solve wide-spread geotechnical tasks related to the deformation and the loss of the bearing capacity of existing buildings and structures.

Although the cost of the injection techniques, compared to different other techniques used for ground modification is high, the practice shows that the injection techniques might be the only feasible solution and provide the most effective ways for many foundation problems, especially in the existing buildings and structures where traditional techniques are limited to use due to various reasons [3]. One of the most efficient injection methods used in this field is soil injection by an expandable resin.

This article aims to provide a comprehensive understanding of the soil injection technology using an expandable polyurethane resin, the behavior of this resin in the soil massive after the injection process, and provide necessary data for practical applications, exploring the advantages and limitations, and possible future development.

The technology has many advantages in field applications and provides a vast solution for several geotechnical problems. The key feature of this technology is that it can be used both for rapid and strictly controlled lifting the foundations with compensation for the resulting deformations and strengthening the foundations, which provides fast technical, modern, and non-destructive solutions under various geotechnical conditions [64,65,66,67,68].

Moreover, the injected resin quickly gains strength. Its use is possible for all soil types since the polyurethane composite does not contain particles, which could be limited by the size of the porosity of the treated soil. The ease of injection, the high mobility, the lightweight of the resin, and the independence of the physic-mechanical properties of the resin from the water level allow this technology to be used in various geotechnical conditions [64,65,66,67,68,69,70,71,72,73]

While adequate research and case studies of the application of this technology have been conducted worldwide, each research has emphasized only some of the technology’s practical applications, such as the resin properties, the effect of the resin on certain soil types, and the composite (soil-resin) modified properties after the injection process [74,75,76]. Generally, the previous studies support a common conclusion: the examined polymer represents a good grouting material with excellent comprehensive performance and is used extensively in geotechnical engineering [74,75,76].

Nevertheless, thus far, few reports could be found on employing this technology for both tasks strengthening and settlement compensation. Therefore, there is no comprehensive understanding of the technology yet that gathers all necessary data leading to a better recognition for this technology in the theoretical understanding and the practical applications [74,75,76].

In this article, the existing data in the literature regarding this technology are studied extensively. As a result, the injection process, the resin durability, the composite (soil-resin) modified properties, the spread and mechanism of the resin in the soil massive, the resin consumption and its proportional dependence on the degree of expansion, the technical advantages are analyzed in detail, discussed, and highlighted.

## 2. Soil Injection Method Using an Expandable Polyurethane Resin

### 2.1. General Description

One of the most recently developed techniques used for soil stabilization and foundation restoration is the soil injection technology using an expandable polyurethane resin [64,65]. The technology was used for the first time in the year (1996) by (Carlo Canteri) the founder of Uretek company in Italy [77]. Since then, non-water polyurethane polymerization methods with different characteristics and high-pressure injection techniques have developed rapidly [76].

The technology is primarily based on injecting the soil massive up to the required treatment depth by two components of polyurethane solutions (a polyol and an isocyanate) that are mixed in a hydraulic system and pumped under a high pressure into the soil, using an injection pistol through a small pre-made hole in the foundation, the diameter of which ranges within (12–30 mm) [64,65,67,70,71,72,77,78,79]. The mechanism of this technology differs from other techniques due to the natural chemical reaction of its components, which leads the resin to expand volumetrically within a specified expansion ratio. As a result, the resin can expand in the soil massive during the injection process up to 30 times its original volume [67,69,78].

The expandable resin is produced by the chemical exothermic reaction between a polyol and an isocyanate when combined in volumetrically established proportions. During the chemical reaction, carbon dioxide is produced, which causes the volumetric expansion of the mixture. The resin’s mixture changes from a liquid to a solid and hardens rapidly (in a few seconds). The reaction time, which depends on the type of the injected resin and the catalysts used, is controlled by the admixed components temperature [79]. By regulating the mixing temperature, it is possible to control the reaction time, which has a significant role in the practical applications of the technology [67,80].

The resin components react immediately, and polyurethane foam swells rapidly, generating a tremendous expanding power. Under an expanding force, it fills the voids and compacts the surrounding medium [76].

### 2.2. The Injection Process

The injection procedure of the technology is explained as the following [64,79]:

- Drilling holes. The diameter of each ranges between 12–30 mm. The diameter of the holes is various according to the equipment and the injection pistol used for the injection process.
- The injection tubes (steel or copper) are inserted into the soil zones up to the depths required to be treated. The injected zones are chosen according to different aspects, such as the soil type, groundwater level, and effective foundation depth.
- The two components of an expandable polyurethane resin (with a high coefficient of expanding) are mixed in a hydraulic system and injected, using a particular injection pistol, into the soil’s massive incrementally through the small holes prepared in advance.
- The injection process is controlled and monitored until reaching the desired results using various developed procedures. The easier and most effective monitoring is using a laser level with high accuracy (0.01 mm).

The resin is injected into the soil’s massive in a liquid state. The chemical reaction of the two components occurs instantly, causing the resin’s expansion by increasing its volume. The chemical reaction begins and ends quickly, and the resin starts hardening in the soil massive within a few seconds (up to 30 s), changing from a liquid to a solid-state, and reaching its final physic-mechanical characteristics within a few minutes (15–30 min) [64,65,67,79,80]. The resin has an expansion pressure potential that can reach up to 10,000 kPa [64,67,69,78].

The resin injection is usually made in successive ‘shots,’ allowing the resin to expand between the shots so that the lifting effect of each shot can be assessed before the next shot is made. As a result, the lift occurs incrementally, which allows for controlling the foundation lifting to the desired level; the foundation’s over lifting risks are prevented. The interaction between these several shots forms a heterogeneous material consisting of several layers [64,65,66,67,69].

The design objectives of the traditional injection techniques are controlled mainly by the type and quantity of the components present in the mixture of the injected material, the injection pressure, and the available volume, which determines the area of influence of the compaction. However, injecting an expandable polyurethane resin is different due to the synthesis chemical nature of its components. The expanding resin does not require an injection of external pressure, and the expansion process occurs due to the chemical reaction of the resin components [67].

In practice, the reaction time depends on the particular resin and catalysts used for the chemical reaction; however, it is also influenced by the admixed temperature of the components. Therefore, controlling the temperature of the components during the injection process allows one to speed up or slow down the reaction time, controlling the speed of the hardening process [67,79,80]. The primary role of the injection pressure is to control the flow of the material, allowing the resin’s propagation through the cracks made by the fracking process. When the resin penetrates the treated foundation’s soil, it increases in volume, propagating through existing cracks and the cracks created by the hydrofracking mode, due to the high injection pressure. The best practice for controlling the lifting process is controlling the ratio of the components mixing temperature and the injection pressure. Furthermore, when the initial lifting is observed, it substantiates that soil strengthening has occurred [64,79]. As a result, the resin’s expanding action is being directed vertically upward towards the foundation, causing the lifting of the construction built above [69].

### 2.3. The Chemical Properties of the Polyurethane Resin

Polyurethane resin is established in practical applications using two based components, both in a liquid state. The compounds used mainly consist of isocyanate and polyol compounds. Polyol is an expansion agent in the formation of polyurethane and it contributes to its volumetric expansion. On the other hand, the isocyanate is a bonding agent in the formation of polyurethane. It contributes to the density and strength of polyurethane. Increasing the isocyanate mixing ratio leads to an increase in the strength properties of the gained resin; however, the consumption of the resin is increased as the expansion ratio is decreased for a fixed injection volume. Thus, the ideal mix ratio is 1:1 because it provides adequate expansion and compressive strength [81]. The resin expansion ratio is mainly controlled by the mixing ratio, resin weight, and existing volume. In a specified injection environment, the expansion ratio is controlled by the weight of the resin being injected. Therefore, it allows controlling of the expansion force and resin properties in the composite (soil-resin) required to attain the desired results [67,68].

When the resin intrudes in the soil, it hardens as the reaction between two components occurs. This liquid injection contains a liquid isocyanate compound and a liquid polyol compound, which react together, forming a high-strength solid polymer [82].

When combined in volumetrically established proportions, the expansion process is produced by the exothermic reaction between a polyol and an isocyanate. A large amount of carbon dioxide is generated during the chemical reaction, which leads to the mixture’s volumetric expansion, forming a spongy structure where the gas bubbles are enclosed. The presence of water that reacts with the isocyanate group is necessary for the production of carbon dioxide. A chemically inert swelling agent, with a low boiling point, is used in the absence of water. This agent evaporates, consuming some of the heat from the polymerization. The resin’s mixture changes from a liquid to a solid-state and solidifies in few seconds [70].

The chemical formation of expanding polyurethane foam is given as follows [83,84,85]:

R–NCO + HO–R’= R–NH–CO–O–R’

Iscocynate + Polyol = Polyurethane

An isocyanate is a functional combination consisting of nitrogen, carbon, and oxygen with the formula R–N=C=O. The oxygen and nitrogen atoms are negative ions, the role of which is imparting the carbon’s electrophilic nature. Isocyanates are known to be a highly reactive chemical. Isocyanate reactions are sorted into two main groups: its reaction with compounds that contains hydrogen reactive, forming an additional product, and the polymerization of isocyanate. The chemical reaction between the Isocyanate and polyol compounds forms a urethane group. The reaction between the Isocyanate and amines forms urea [84].

Polyol (–OH) is an organic group containing hydroxyl groups, an interfering ester, an ether, an amide, an acrylic, and others. Polyester polyols are most commonly used for the reaction in the production of polyurethane resin. They are prepared by the reaction of glycols, which also consist of ester and hydroxylic groups. The properties of PU depend on the molecular weight of the cross-linked polyester polyols. Some polyester polyols result in rigid PU resin/foam, which has good heat and chemical resistance. High-molecular-weight polyols yield flexible PU resin/foam, while low-molecular-weight polyols produce rigid PU resin/foam [85].

Due to the severe toxicity of isocyanate, some researches have been conducted to reduce the use of isocyanates to synthesize polyurethane. Non-isocyanate-based polyurethane foam has been industrialized from sustainable resources, considering a new class of polyurethane polymers. The new polymer class allows for mitigating health and environmental concerns [86,87].

### 2.4. The Density of the Injected Resin

The solidified resin’s density formed in the soil’s massive, after the injection process, is the most significant factor that affects its mechanical properties, thereby identifying the degree of the soil strengthening required after the treatment. The resin’s density varies due to its expansion properties, and the amount of the resin used plays a role in the final density formed. Thus, injecting a different amount of the resin in a constant equivalent volume leads to different densities than its initial density in the liquid state, although the amount of the injected resin, the mixing temperature, the injection pressure, and the soil type play a significant role in the resin propagation and expansion processes. However, the final resin density established in the soil’s massive is unpredictable without conducting laboratory investigations. Nevertheless, in a homogeneous injection environment, the density is controlled primarily by the amount of the injected resin. Thus, the density of the injected resin could be predicted using a reference injection environment; thereby, it can be controlled by the amount of the injected resin relatively, considering the resin’s expansion ratio [67].

The compressive strength correlates with the test sample’s unit density directly. The compressive strengths of the hardened foam material are dependent on several factors and conditions during the injection. Coupled with other factors, such as the duration of injection time, volume of material injected, soil and air temperatures, and the soil’s resistance to compression, the path of the injected foam becomes unpredictable. Regardless of the path the injected foam material takes, it is essential to adjust the controllable factors to maximize the density of the injected foam and the resultant compressive strength. The compressive strength test results show that injecting more material until a bump is observed on the surface allows increasing foam strength formed in the soil [72]. It is justified by increasing the resin density in a steady injection volume, thereby decreasing the resin’s expansion ratio [67].

The pressure exerted during the expansion process, generated by the chemical reaction of the components and the subsequent density of the PU, depends on the extent to which the gas in the bubbles can expand before polyurethane foam hardened [70].

The Density of the components in the liquid state is equal to 1070 kg/m^3^, which is approximately equal to the water density. Therefore, under these conditions, the expanding volume is equal to 30 times the original volume of the mixture [78].

Different polyurethane grouts yield differing densities, shear strength, and permeability. The greater the expansion, the lower the strength because the expansion pressure develops, and the final density depends on the confinement level. For a foundation remediation application, an expanding resin that hardens within a few minutes is desirable so that its effect can be evaluated immediately after the injection [88]. In addition, the resin is only sensitive to UV light and some synthetic chemicals that are not usually found in the foundation soils; therefore, it is considered to be stable [70].

Based on the mechanical properties of polyurethane foam, presented in the study of Keene [89], polyurethane foam has at least two potential applications. First, when stabilizing ballast from the bearing surface of the tie-down to the sub-ballast layer, the compressive strength results and resistance to accumulation of plastic strain indicate that these areas can have a much longer life cycle than untreated ballast. Second, when stabilizing ballast at the base of the ballast layer (i.e., as an underlayment), the flexural strength results indicate that Polyurethane-Stabilized Ballast PSB can withstand loading while preventing intrusion of fines and water from the sub-ballast and subgrade layers. However, data and performance from the actual field installation of PSB is still needed for validating the laboratory results [89].

Determination of the resin density is essential for the foam’s mechanical characteristics; as the foam’s density increases, the strength, hardness, and resistance to fatigue increase. Consequently, polyurethane may possess a density that would be sufficient in the case of rigid foam, but high open-cell content would result in mechanical properties that are substandard for the intended design of rigid foam.

The resin was tested in the rise direction by Valentino et al. [90]. The stress-strain responses were established to determine the peak stress, Young’s Modulus, and fracture energy densities, whereby the increase in the density increases the failure stress and Young’s Modulus; however, the yield strain was reduced. These results are consistent with the test results of Saha et al. [91], which indicated that the peak stresses are strongly dependent on the density of PU. In particular, an exponential relationship between the peak stress and resin density and Young’s Modulus and resin density were found. The experimental tests result, which were performed on two different types of resin, revealed that the higher the confinement stress is during the expansion phase, the greater the hardened resin density.

The water absorbance of closed cell rigid polyurethane foam is typically governed by the density of the foam. The study has shown that the water absorbance relationship is directly associated with the foam’s achieved density. The foam used in this study was typically 70 kg/m^3^ to 90 kg/m^3^. This range is on the high end of the spectrum, but there is expected to be high strength and low water absorbance. The foam strength is directly related to the density formed compared to the solid density of the constituent polyurethane. Polyurethane’s physical and mechanical properties depend on its density, as PU foam exhibits different behaviors proportional to the density [92].

The resin does not add additional weight to the soil; moreover, the density of the treated soil is increased because of the additional volume of the injected expandable resin; however, the injected resin’s weight is relatively small. Therefore, no extra settlement in the layer beneath the injection occurs [64].

**Figure 1:**
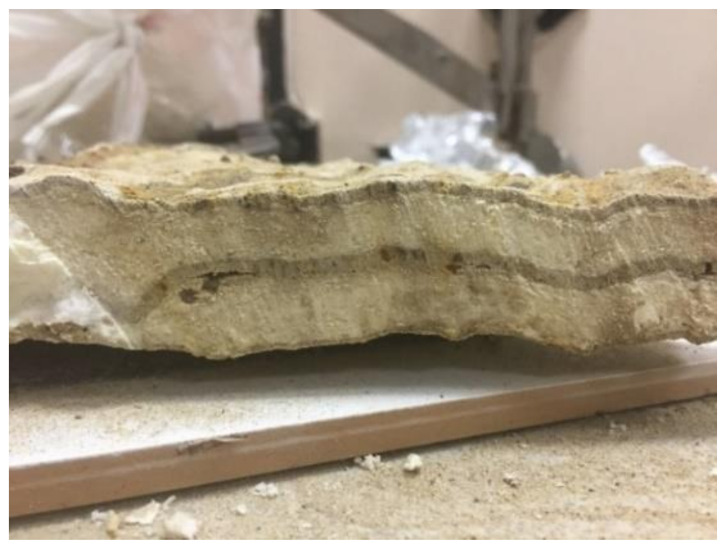
Resin samples were extracted from the injected plot after the injection process of the field investigations [67].

Sabri M.M. [67] investigated the density of the resin formed in the massive injected soil. During the field investigations, four resin pieces were extracted from different locations of the injected site to determine the average density of the resin formed after the injection process, as shown in Figure 1.

It was found that, in cohesionless soils, under a certain average injection pressure of (100 bar) and mixing temperature of (15 °C), the average density of the resulting resin formed in the soil’s massive is 0.184 gm/cm^3^.

Sabri M.M. [67] stated that the resin density in the liquid state is 1.1 g/cm^3^. The resin’s density, which has been formed in the massive of the investigated soil, after the injection process of the field experiment compared to its density in the liquid state confirms that the resin was expanded six times its original volume. According to the resin consumption determined during the injection process (paragraph 2.6.), it was found that the volume of the resin required to strengthen the soil was 4% of the total volume of injected soil, while 2% was used to raise the foundation to the predetermined level of 1 cm. Changes in the volumetric proportion of the resin components lead to control of the expansion ratio and the final resin density, consequently regulating the expanded resin’s final mechanical properties. Sabri M.M. recommends this procedure to calculate the composite’s soil-resin final strength and stiffness after the injection process, determining the modified soil properties under reference injection conditions [67,68].

### 2.5. The Propagation of the Polyurethane Resin

The resin propagation during the injection process depends on many factors such as the injection pressure, component admixed temperature, the type of the soil, and the amount of the injected resin.

There are two main propagation theories proposed during the injection of the expandable resin into the soil massive:

#### 2.5.1. Cavity Expansion Theory

Although some conducted studies stated that the flow and propagation of the PU expandable foam are subjected to the cavity expansion theory when injected into the soil massive [77,78,80,93,94]. However, this theory is considered if the resin is injected at low injection pressure, leading to the resin expansion, not along the fracture but in the form of a ball or cylinder; consequently, it leads to high resin consumption and the inability to achieve a uniform distribution for the foundations lifting, and it turns out to be ineffective.

According to Favaretti et al. [78], the injected resin permeates the soil following the weakest path. In the case of cracked soil, the resin fills and expands the existing cracks. According to the laboratory observations, the resin penetrates cracks of 0.1 mm, although the resin does not expand very far in these thin cracks.

According to Buzzi [69], when injecting the foam into a desiccated clay, the resin can either propagate through existing cracks or create new fractures in the soil, propagating toward the surface of the foundation. Thus, the foam is incrementally injected into the soil, and its expansion is used to lift settled structures. With the foam following the weakest path, its propagation is a somewhat random phenomenon. Moreover, the foam hardens in veins, which can be of various morphology and dimensions. Generally, the wider the cracks, the further the resin can propagate. The development of foam dendrites at the soil/foam interface, to a depth of about 3 mm, was also noticed. A layer of cells is simply in contact with the soil without being bonded to it. A dendrite forms if a soil’s void is larger than the size of the existing basic cell at the crack interface. Some soil particles were found within the resin in the vicinity of the soil/foam interface. However, very few soil particles were found in the bulk of the foam, i.e., in the middle of the vein. When it propagates in open cracks, the resin interacts with the soil only at the interface. Buzzi stated that the formation of macrovoids is allowed by the significant width of the crack (20 mm), and they are not so apparent in foams formed in smaller cracks. However, when precisely looking using the SEM, it appears that the foam in more minor cracks is still heterogeneous. The result of the propagation and hardening of the foam is the distribution of the cell sizes. The cell size distribution does not appear to be uniform or gradual, although there is some suggestion of gradual changes in orientation.

As a part of the resin propagation study, large injected and non-injected specimens were collected using a 300 mm diameter push-pull tube. These specimens were used to perform swelling tests in the laboratory. The results have shown that the resin’s diffusion is relatively unpredictable and that injected resin cannot prevent hydration in an injected soil but can, at most, delay it. However, the laboratory and in situ tests showed that the resin-injected expansive soil does not exhibit an enhanced swelling potential, probably because a significant number of unfilled cracks remain in the injected soil, which provides sufficient relief in the swelling soil to prevent the injected soil mass from swelling excessively. Based on this understanding and the observations of this study, it is suggested that, by injecting deeply (that is, below the depth of cracking), the resin is likely to fill relatively few of the cracks during injection so that a significant amount of voids can still be expected in the soil mass. The author stated that “Consistent with the literature results, the swelling pressure of the soil is then expected to be much lower than that usually measured in the laboratory under total confinement” [70].

Based on Buzzi’s [70] observations, two propagation and lifting mechanisms were identified and summarized: If the injection occurs within the cracked zone, the resin is likely to intercept and propagate through existing cracks as it expands. In this case, it forms a smaller body near the injection point, and it often reaches the surface, allowing it to act directly on the structure. It has been observed that, even if the resin propagates extensively through cracks to reach the surface, crack filling is still a localized phenomenon, and many of the cracks around the injection remain unfilled. Alternatively, if the resin is injected below the crack depth, the resin creates a larger body at the injection point and fills and propagates through relatively few cracks. Thus, it is unlikely to reach the surface; instead, it can lift the cracked, overburdened soil and any overlying structure that may be present. This ability to lift at depth is due to the significant expansion potential of the resin, which can fracture the soil at the injection point if no major void is present.

In 2012, Fityus et al. [71] carried out a laboratory experiment to study the effects of resin injection on the expansiveness of cracked expansive clay. The characterization of soil mass behavior, where inhomogeneity exists on large and many scales, is challenging. While full-scale field tests afford the best solutions, laboratory-scale assessment can also provide valuable outcomes, provided the samples are large enough in both size and number to take account of the inherent variability. The results indicate significant variability in the swelling behavior of the Maryland soils, even when tested with large samples. However, it was considered that 300 mm diameter samples were sufficiently large to represent the injected expansive soil, based on the resin distributions.

Further, Fityus stated that “despite the scatter in the results, the significant number of results gives sufficient confidence to conclude that resin injected samples are not prone to exacerbated swelling, all other things being equal”. Indeed, if a conclusion were to be drawn, it would be that resin injection seems to reduce the potential for soil swelling, contrary to the intuitive expectation that the soil is forced to displace more in the vertical direction by filling the cracks. There is no obvious explanation for this, according to Fityus’s opinion. A possible explanation is that, although the resin intrudes and fills the larger cracks, it does not seem to travel far into the smaller (<1 mm) cracks. So, as a first consideration, not all of the crack space is eliminated by resin injection. Beyond this, there is also likely to be a secondary effect of new crack propagation; the larger cracks are forced apart by the expanding resin. The net result may be that, while the larger cracks are filled by resin, their lost volume is compensated for by creating a new population.

Nowamooz [93] attempted to develop an analytical analysis for cylindrical pore cavity expansion in cohesive frictional soils, obeying the Mohr–Coulomb criterion, applied to simulate the pressuremeter tests before and post-injection. The model parameters were calibrated by a constant value of the elasticity parameters and the soil’s friction angle before and after injection. As a result, a significant increase in cohesion parameters was observed due to soil densification and due to resin expansion. However, further points, such as the optimum injection volume and its propagation in clays, the unsaturated state of clay soils before injection, or the modification in the clay structure after injection are still necessary to consider to complete this study [93].

The resin’s propagation capacity in the different soil types can be established according to the following criteria. In gravels and sands, the propagation is mainly by impregnation of the intergranular spaces. On the other hand, in clays, the voids and fractures present in the soil’s macrostructure are saturated by the resin or compressed by the swelling pressure during the expansion process [80].

Valentino [95] investigated a relationship between the density of two polyurethane resins and confinement pressure under expansion conditions and found an exponential relationship between the peak stress and resin density and between Young’s Modulus and resin density. However, the diffusion behavior of polymer grout materials in the soil was not considered.

#### 2.5.2. Propagation in the Hydro Fracturing Mode

Popik [72] stated that the injection process tended to form a honeycomb structure type lattice within the peat layer. The formation and appearance of the lattice structure would indicate flow through paths of least resistance (hydraulic fracture type process) rather than mixing within the structure/matrix of the peat. It was observed that, in areas where the volume of material injected was increased, the lattice walls were thicker, resulting in smaller ‘peat cells’. This lattice structure has the potential to add stiffness to the peat layers. According to Popik, further research should be considered to refine the injection process to produce an improved lattice structure.

Sabri M.M. [64,65,66,67,68] has established and proved that, when the resin is injected under high pressure (average of 100 bar), the resin expands in the soil’s massive propagating in the mode of hydrofracturing rather than the cavity expansion mode. This outcome was obviously seen through a series of field and laboratory investigations, justified by an established Finite element model that predicts the settlement and bearing capacity of the composite soil-resin after the injection process under a homogenous injection environment [64,65,66,67,68]. Further, the resin’s diffusion patterns in the soil massive were investigated, identified, and set as a reference through which the average configuration pattern of the resin in the soil massive was established.

**Figure 2:**
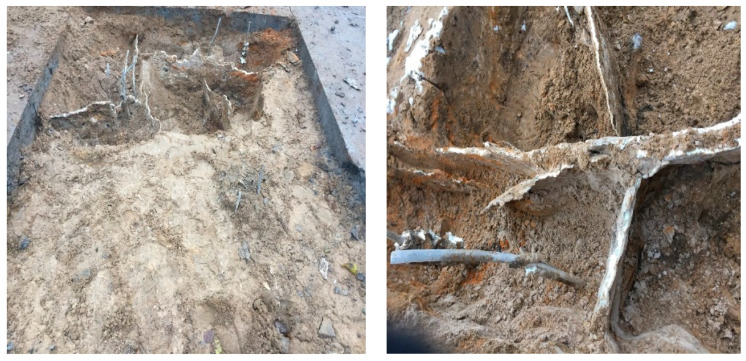
Different resin sections were distributed in the massive of the injected soil at a depth of 0.4 m [65,68].

**Figure 3:**
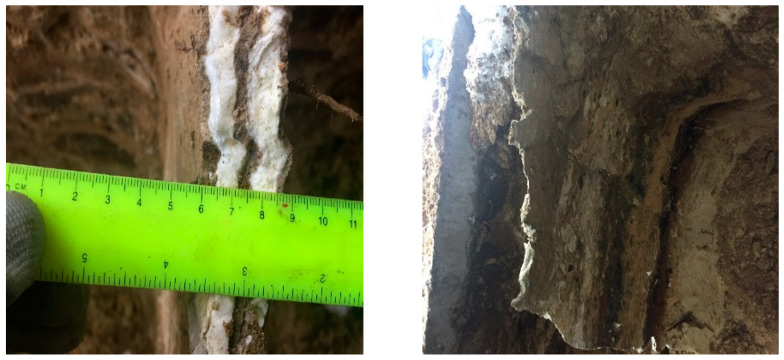
Different resin sections were distributed in the massive of the injected soil at a depth of 1.1 m [65,68].

Although the propagation of the injectable resin is relatively variable, based on several factors such as the type of the soil, the injection depth, and the amount of the resin components, which can form different density resin based on its expansion ratio in the soil massive; nevertheless, under a homogenous environment, the injected resin diffuses in the non-cohesive soil, forming identical continuous walls of foam plates along the whole injected depth, surrounding the injected soil from all sides, connecting at the edges of the resin within a distance interval around 30–50 cm, and the average thickness of the resin is 1–2 cm, approximately, as measured. Figure 2 and Figure 3 show different sections of the resin propagation in the massive of the investigated soil. Consequently, the soil and resin form a composite system of the elastic perfectly plastic environment, based on which a FEM model calculates the soil’s bearing capacity and the settlement after the injection was established [68].

The peculiarity of the two-component injection technology is the very fast solidifying process of the resin. During the solidification process, the pores of the sand are calmed, and the injection material spreads in the form of hydraulic fracturing made by the injection pressure. Thus, the resin’s diffusion through the pores, due to the filtration of the soil, practically does not occur [68].

**Figure 4:**
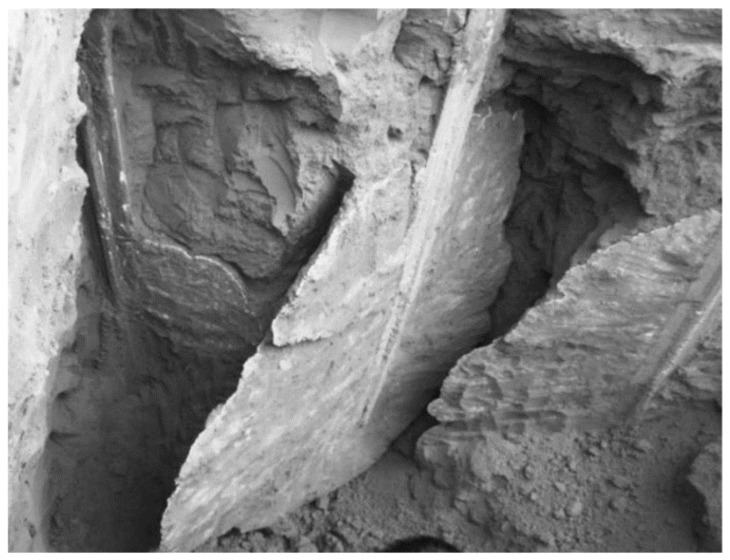
Solidified polymer segment [76].

Guo, Chengchao [76] investigated the resin diffusion in silty loam soil. The investigation results enhance the propagation outcome made previously by Sabri M.M. [64,65,66,67,68] in non-cohesive soils and are consistent with it. The results confirm that the polymer materials perform sheet fracturing diffusion in soil rather than complying with the conventional theoretical assumption for grouting spherical or columnar diffusion, and it is more akin to splitting grouting. When the polymer materials are injected into the borehole, they expand immediately under a chemical reaction and vertically split the soil along the borehole wall. The polymer grouts intrude into the fractured fissure, and under the expanding power of the polymer, the crack further extends until the grout cures. Finally, the solidified polymer material forms a somewhat oval segment in the crack. The heights of all segments are equal to the length of the enclosed part of the grout hole, and their width increases as the amount of polymer grout injected is augmented. The polymer segment cross-section is approximately wedge-shaped, with a maximum thickness of 2.5 cm near the grouting hole and a minimum of 0.8 cm on the edge of the segment (Figure 4). The results also reveal that the polymer materials have limited osmosis and adhesion effects on soil. Its permeation results in a thin transitional cementation zone between polymer and soil.

#### 2.5.3. Conclusion of the Resin Propagation in the Soil Massive

Based on the above, it could be concluded that, in a homogenous injection environment, the resin is subjected to three propagation mechanisms in the soil’s massive:

1. The resin’s diffusion in a hydrofracturing mode leads to forming solid resin plates in the massive of the injected soil with a specified length, depth, and thickness. Consequently, a composite of soil-resin is formed, where the resin serves as a bearing element, strengthening the soil and lifting the overlying buildings and structures. The role of the injection pressure is to control the flow of the material in the soil’s massive. The peculiarity of the two-component injection technology is the rapid solidifying process of the resin. During the hardening process, the pores of the sand are calmed, and the injection material spreads in the form of hydraulic fracturing made by the injection pressure. Thus, the resin’s diffusion through the pores, due to the filtration of the soil, practically does not occur
2. The resin diffuses through the existing pores and cracks due to the filtration of the soil; however, it is limited by a few centimeters of dendrites around the injection zone and has no significant strengthening and lifting results. However, the resin’s diffusion through voids, by the filtration of the soil, practically does not occur due to the high injection pressure.
3. A cavity expansion mode, if the resin is injected at low or no injection pressure, leads to the resin expansion not along the rupture but in the form of a ball or cylinder; consequently, it leads to high resin consumption and the inability to achieve uniform distribution for lifting foundations and turns out to be ineffective.

### 2.6. The Consumption of the Resin

The actual consumption of resin to lift the foundations to a certain level and strengthen the soil beneath the desired parameters has been investigated. The actual resin consumption and its volume, as a reference of the injected soil massive, were established by Sabri M.M [65,67,68], where the net amount of the injected resin consumed for lifting a specified load to each 10 mm, strengthening the soil beneath, was registered by 180 L, which is equal to 1% of the whole volume of the injected soil. The amount of the injected resin was recorded at each injection point at two stages during the injection process. The first stage is the consumption required for the soil strengthening process till the initial lifting occurs and is measured. The second stage measures the resin consumption required to lift the foundation, following the soil strengthening process. As a result, the resin consumption required for soil strengthening was recorded at 123 L, while 57 L were consumed to lift the foundation to 1 cm. Thus, it was concluded that the resin required for the soil strengthening is more (almost twice) than the amount required for the lifting process. Therefore, in this particular study, it was concluded that the optimum injection volume for non-cohesive soils is 1% of the total soil volume (10 L for each 1 m^3^ of the soil) [65,67,68]. Table 1 shows the resin’s consumption at each injection point as recorded in-situ during the injection process [68].

### 2.7. The physic-Mechanical Properties of the Soil-Resin Composite (Modified Properties): Field and Laboratory Investigations Data

For geotechnical engineering purposes, understanding the physic-mechanical and hydro-mechanical behavior of the modified soil, after the injection process, is essential for practical applications. As explained above, in a reference injection environment, the physic-mechanical properties of the resin, and the degree of its expansion, depend mainly on the final density of the expanded resin. Consequently, the degree of soil improvement directly relates to the resin’s final mechanical properties formed in the soil’s massive. Since the necessity of accurate data that investigates the resin’s mechanical properties, based on the degree of its expansion, is controlled by the amount of the injected resin, it is a significant task. Such data allows predicting the necessary mechanical properties to determine the field applications’ desired lifting and strengthening results [67,68].

#### 2.7.1. Laboratory Investigations Data

In 2008, Buzzi [69] conducted a laboratory experiment to investigate the polyurethane expandable resin’s structural, mechanical, and hydraulic properties in a typical clayey soil using Scanning Electron Microscopy and unconfined uniaxial tests. The density of the studied foam in this experiment ranged from 37 to 145 kg/m^3^. It was found that the yield stresses in both transverse and rising directions are similar and ranging between 250 and 500 kPa, and the compression stress ranges from (30–40 kPa). The pressures of up to 10 MPa have been reached. A reduction of 68% of the clay peak swelling pressure has been recorded for soil with as little as 1% macro voids in its total volume. Furthermore, the author stated that the foam is relatively resistant to water absorption, and it can be used to exclude water in many geotechnical applications, and the quality of the foam formation is not affected by the surrounded water.

In 2014, SAI TEJASWI LANKA [88] conducted a laboratory experiment to study the effect of different surcharges on the vertical displacement and the compressive strength of the resin. According to SAI TEJASWI LANKA, this study has shown that the value of the resin compressive strength is independent of the surcharge load, and the value of the resin vertical displacement increases relatively with the amount of the injected resin.

In 2015, Norbaya Sidek [96] conducted a laboratory experiment to study the properties of sand modified by polyurethane resin. Different samples were tested using the unconfined compression test. The experiment showed that the compressive strength of the modified sand is higher than the regular sand tested, considering increasing the soil’s properties values such as the moisture content, the particle size distribution, and the specific gravity.

In 2016, Fakhar [97] conducted a laboratory experiment to compare the expandable polyurethane resin, used to compensate for an operational highway, and the geocrete method. Soil-resin cores were extracted from a highway after its injection by the resin. The laboratory investigations of these samples have shown that the injected foam successfully filled all existing voids and cavities, whereby the swelling index reduced impressively.

In 2016, Iman Golpazir [92] conducted a laboratory experiment that studied the dynamic behaviors of Polyurethane foam-sand mixtures utilizing stress-controlled cyclic triaxial tests. These laboratory tests were performed to evaluate the possibility of using PU foam as an appropriate material to reduce seismic earth pressures against geotechnical structures such as retaining walls, bridges abutments, and buried pipes. The results of this experiment showed a significant improvement of the dynamic shear modulus of the sand-foam mixture. An increase in the polyurethane resin percentage in the mixture leads to an increase in the shear resistant of the sand consequently. The evaluation of the dynamic properties of PU injectable foam indicates that this material can be considered as a potential alternative for the reduction of seismic earth pressures.

In 2019, Kumar A et al. [98] studied the effect of polyurethane resin on black cotton soil. Results showed a significant increase in unconfined compression strength and the CBR value of the subgrade. Besides, the voids in the soil had been filled and covered with foam.

RPF injection techniques were initially developed to remediate differential settlements in foundations beneath structures. In 2016, George A. et al. [99] stated that “the subgrade quality significantly impacts both the initial cost of the railroad structure and the subsequent maintenance and outage costs; however, its practicability for stabilizing subgrade will have to be evaluated further in the laboratory and field on various soil types.”

In 2019, Jais I [100] investigated the compressibility of peat soil improved with polyurethane foam. The oedometer test results have shown a significant improvement in the compressibility parameters, such as void ratio, compression index, and swelling index of the peat soil after treating the soil with the polyurethane resin.

**Figure 5:**
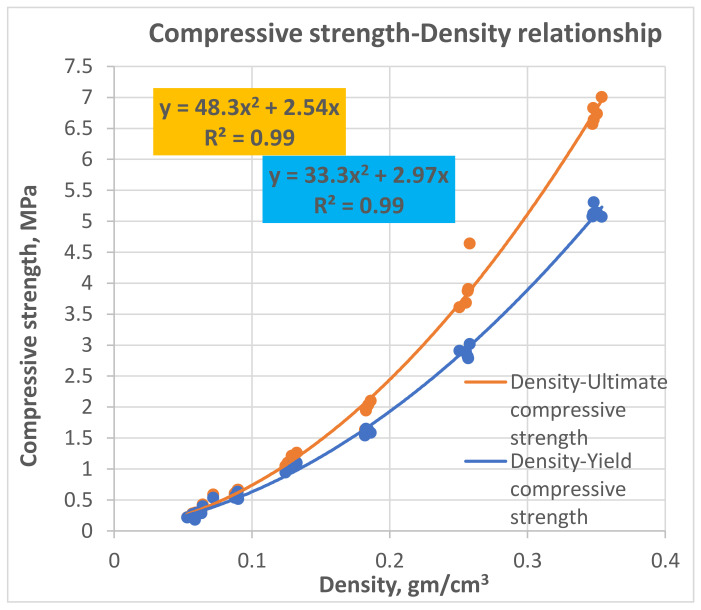
The relationship between the resin’s density and its compressive strength [67].

**Figure 6:**
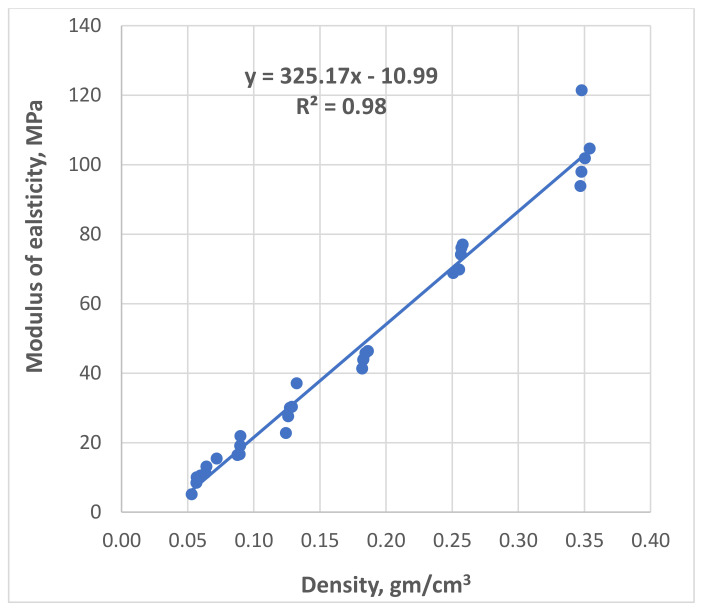
The relationship between the resin’s density and its elastic modulus [67].

**Figure 7:**
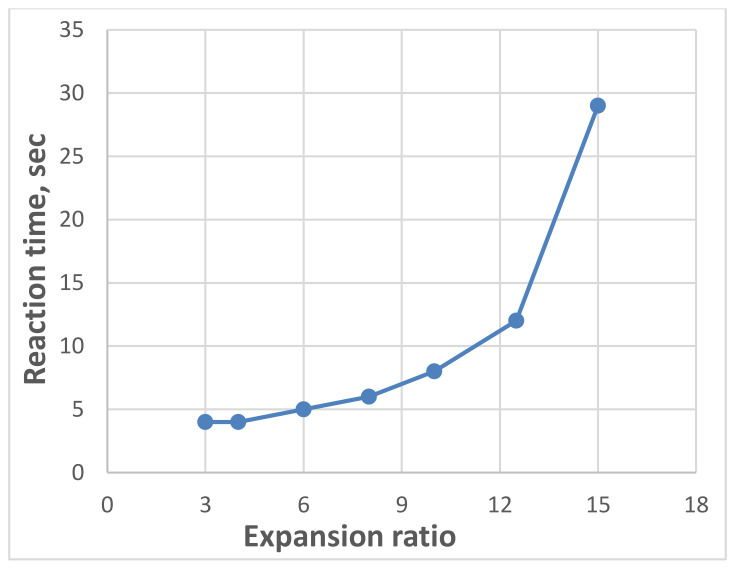
The reaction time and the resin volumetric expansion relationship [67].

**Figure 8:**
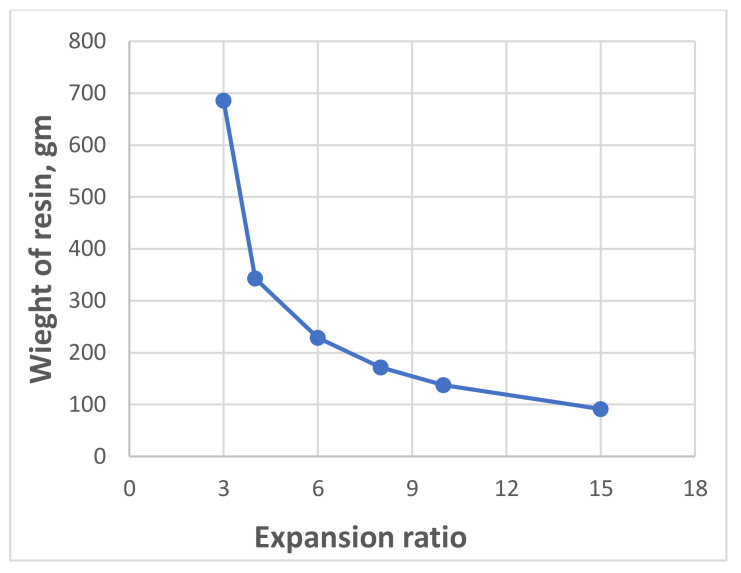
The relationship between the volumetric expansion ratio and the weight of the resin [67].

In 2020, Sabri M.M. [67] investigated the mechanical properties of the expandable resin, based on its volumetric expansion ratio, controlled by the amount of the injected resin. According to the results of laboratory experiments, the resin’s strength-density and Young’s Modulus-density relationships under compression within density ranges (0.053−0.354 gm/cm^3^) and volumetric expansion ratios (3–15), respectively, were revealed, as shown in Figure 5 and Figure 6. It was found that the reaction time of the injected resin increases in direct proportion with the weight of the resin; however, the relationship is non-linear, as shown in Figure 7. It was also proven that the volumetric expansion of the injected resin decreases in direct proportion with the weight of the resin; however, the relationship is non-linear, as shown in Figure 8.

The relationships obtained by Sabri M.M. proved the dependency of the mechanical properties of the expandable resin on its density, which variated due to its volumetric expansion properties. The obtained results show that the proposed resin can be formed in various densities, allowing a wide variety of the mechanical properties in the soil massive up to (*E* = 8–73 MPa, σ = 0.5–4.6 MPa), respectively, at expanding ratios of ranges (3–15 times). Consequently, this resin is considered a high-strength injected material compared to various injection materials used for soil stabilization, considering the rapid lifting and strengthening processes. The resin’s fluidity in the hydrofracturing mode allows its propagation in different soils regardless of their porosity size. The obtained relationships play an essential role in the theoretical and practical applications of the injection process to determine the soil modified properties and the necessary consumption of the resin required for given soil strengthening and foundation lifting if combined with an adequate finite element model.

In 2021, Al-Atroush et al. [101] performed an extensive laboratory experiment to assess the efficiency of the closed-cell hydrophobic polyurethane foam in mitigating the high swelling and shrinkage response of montmorillonite content Na-bentonite clay. The results have shown that, even after several wet-shrink cycles, the polyurethane resin effectively reduced the swelling and shrinkage potential of the reactive expansive soil.

#### 2.7.2. Field Investigations Data

##### Swelling and Permeability Tests

Further study by Buzzi, in 2010 [70], was a field application to evaluate the laboratory experiment conducted previously and to investigate the effect of the injectable resin on the permeability, the hydraulic conductivity, and the effect of the resin on the natural swelling of expansive clay soil located in Maryland, Australia. It was found that the injected resin does not prevent rehydration of the soil, but it delays it, at most. The permeability of the soil was decreased, according to this study. There are no adverse effects of the expandable resin on the overlying constructions, due to the natural swelling process, as the injected resin is non-toxic, and there is no possibility for any chemical reaction with the soil’s expansive minerals.

##### Dynamic Cone Penetration Test

In 2010, the effect of the resin on the lifting process of road pavement overlaying organic peat located in Florida was studied by Popik [72]. It was found that the earth pressure of the injected resin increased rapidly during the injection process. Moreover, the resin in cohesive soils forms a honeycomb structure, and the Polyurethane formed in peat did not mix with the peat but fractured it. The honeycomb structure of the polyurethane resin has the potential to support and transfer operational pavement loads through the peat layers. Nevertheless, the cone penetration data showed almost no increase in the stiffness of the peat layer after the injection of the resin.

Consequently, in this study, the soil stabilization beneath the pavement failed, according to the DCPT test results. Thus, conclusive evidence of the success of technology in the field of soil stabilization is still needed, according to Popik’s investigations. The author of this study recommended that different approaches and more studies are required to study the potential strength of the foam formed in the soil massive.

In 2011, P.Hellmeier [102] studied the influence of the polyurethane resin grouts on non-cohesive gravel soils and two different types of cohesive soils. An improvement in the dynamic resistance of the soils was noticed through the results of the DCPT.

In 2018, the effect of the injected resin on the mitigation of liquefaction potential and seismic response of a sandy soil, injected by an expandable polyurethane resin, was studied by Traylen [103] utilizing different types of soil investigations such as pressuremeter test, DCPT, Dilatometer test, and PLT. A considerable improvement of the dynamical resistant of the tested sand after the expandable resin injection was observed, accompanied by an increase in the relative density of the tested sand. According to DCPT results, the relative density increased by 30%.

**Figure 9:**
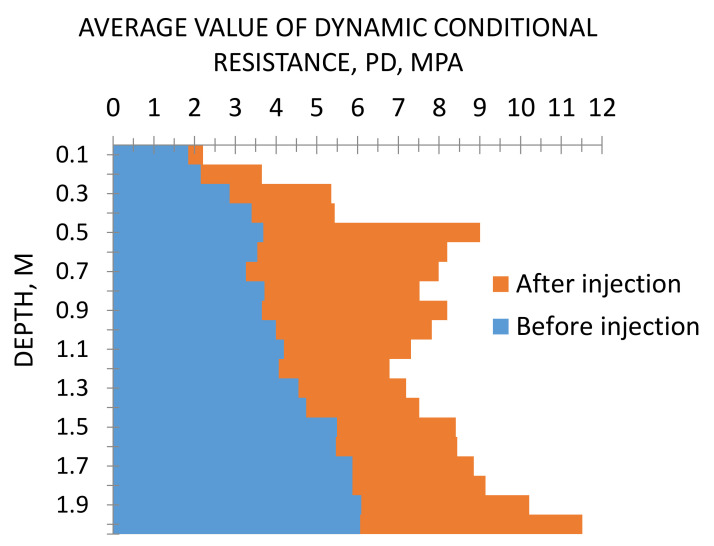
The average value of dynamic resistance of 20 investigated points before and after resin injection [73].

In 2018, Sabri M.M. [64,65,66] carried out a series of field experiments to investigate the effect of the injected resin on the physic-mechanical properties of the non-cohesive soils. Based on the results of field tests of the investigated sandy soil, before and after its injection by two-component expandable polyurethane resin in the hydraulic fracturing mode, it was revealed that: indicators of the dynamic resistance, using the dynamic cone penetration test DCPT, of the investigated non-cohesive soil increased after the injection with the resin. Figure 9 shows the average increase in dynamic resistance is 81%.

##### Plate Load Test

According to P.Hellmeier [102], the calculated deformation modulus, using the plate load test, has been increased by 11% and 43%. However, experiment results have shown that the performance of the resin in the soil massive seems to depend on the soil type, the injected resin type, and the resin’s strength properties.

The plate load test investigations carried out by Traylen [13] have shown a significant increase in the Modulus of Subgrade Reaction (k). The modulus has been increased by around 50 to 90% at different injection locations.

In 2018, Sabri M.M. [65] investigated the properties of the sandy soils before and after its injection by the expandable polyurethane resin. Investigated soil’s bearing capacity increased by 67% and 125% at the investigated depths of 0.4 and 1.1 m, respectively, after the injection. The calculated soil’s deformation modulus increased by 55% and 203% at the investigated depths of 0.4 and 1.1 m, respectively, after the injection.

Consequently, Sabri M.M. [64,65,66] revealed that the deformation modulus depends on the depth, even though the injection of the solution was carried out evenly over the depth and, therefore, the deformation modulus values should be approximately equal at the investigated depths of 0.4 and 1.1 m. Additional research is needed to clarify the changes patterns in the deformation modulus at various depths. Nevertheless, the main advantage of this technology is the possibility of settling sediment—that is, using it as a “liquid jack.” In light of this consideration, settlements are excluded, due to the lifting of the foundations by the presence of the resin, and not due to the changes in the deformation modulus.

##### Dilatometer Tests

In 2015, Niederbrucker [77] investigated the horizontal stresses of the injected resin at three different sites using the flat dilatometer tests DMT. The results have shown a significant correlation between the soil injection using polyurethane resin and the dilatometer modulus. It was found that the expansion of the injectable resin causes an increase in the horizontal stresses of the soil. This increase could be measured and expressed by the dilatometer modulus. The modulus depends on the horizontal distance between the injection point and the point of measurement. The first test showed that the stress influence could be measured up to a distance of about 1.5 m, which expresses the effective influence zone of the resin. However, the effect of the resin on the soil properties in this experiment is not substantiated. Niederbrucker stated that “large-scale experiments and soil investigations are required to study the effect of the resin on the soil properties as the performed flat dilatometer test is an indirect site investigation method.”

According to Traylen [103], the Dilatometer test results showed an increase in horizontal stress index KD from 50 to 150% after the resin injection.

##### Pressuremeter Test

In 2016, Nowamooz [93] attempted to study the effect of the resin on the behavior of high plasticity clay. As a result, an analytical model was provided based on the pressuremeter test results obtained from a field injection. Nowamooz stated that “a better analytical solution and additional laboratory and in-situ investigations are required to determine the behavior of the resin in the soil massive.” According to Nowamooz’s recommendation, further studies to obtain the optimum injection volume, the resin propagation, and the resin’s effect on the physic-mechanical soil properties are necessary to increase the efficiency of the technology in practical applications.

##### Three-Dimension Topographical Electrical Resistivity Test

In 2015, T. Apuani [104] developed a procedure for monitoring the injection process, utilizing the 3D topographical electrical resistivity test, which allows predicting the locations of the injected resin in the presence of water by the variation of electrical resistivity due to the water migration. In this particular study, an increase in resistivity was recorded in the treated volumes, accompanying the changes in the soil properties. In contrast, a decrease in resistivity was observed in surrounding volumes due to the effect of the increase in their migrated water content from the injected volumes. T. Apuani recommended this procedure to monitor and control the injection process, and the resin can be controlled and redesigned according to the results obtained from the electrical resistivity test during the injection process.

In 2015, Warren [105] studied applying the technology in a railway track substructure located in Dayton, Illinois, USA. It was found that the performance of the substructure was increased after the injection of the resin.

In 2016, Alsabhan Abdullah [106] continued the study of Warren. A railroad track subgrade located in Madison, Wisconsin, USA, was injected using the polyurethane resin. The injection was controlled, utilizing the procedure of 3D topographical electrical resistivity. The study has shown that the technology provides adequate lifting and maintenance of the railroad track and the ability of the injected resin to extrude the water from the soil, filling the voids and the cracks. Alsabhan Abdullah stated that “injection appears to be a superior cost-saving than traditional maintenance used in this field.”

According to Traylen [103], the Shear waves velocity, measured by the pressuremeter test, increases by around 50% after the injection process.

From the above, it could be concluded that the expandable polyurethane resin significantly influences the physic-mechanical and hydraulic properties of the treated soils, besides the ability to quickly remediate the foundation of the existing buildings and structures.

##### Case Studies

According to Shi et al. [107], in 2013, the ground of one battery factory workshop in Shanghai city of China was treated with an expandable polyurethane resin. Shi et al. stated that “the floor was leveled to the ideal position, and the maximum uplifting value is about 20 mm.” The ground was stabilized by comparing the deflections tested by FWD before and after grouting. This case study revealed that the expandable resin is reliable for treating the floor with different subsidence [107].

In 2016, Jeremy Hess [108] studied the effect of the injected resin on the groundwater level and hydraulic conductivity. It was found that the injected resin can be used successfully as a groundwater level barrier and to reduce the soil water content. The study showed that the injected resin could be the best practice as an injected barrier.

In 2016, Pavana K et al. [109] carried out a comparison of pavement slab stabilization using cementitious grout and injected polyurethane resin. The author stated that “the in-situ rehabilitation of pavements makes slab stabilization technology an attractive alternative to complete replacement, yet, very little performance monitoring data are available in the literature.”

In 2017, Jais I [83], in his case study, compiled the case studies of rapid remediation using PU resin/grout in Malaysia. The overall results showed that implementing the soil injection technology, using an expandable resin at five different sites for rapid lifting and stabilization, was successful.

In 2020, Diana Che Lat et al. [110] compared ground improvement using lightweight PU foam and cement grouting slab using FEM analysis. The results indicate that the settlement could be prevented using polyurethane expanding resin as a ground improvement method. In light of this consideration, the optimum thickness must be applied to prevent uplift, which can jeopardize infrastructure stability. On the other hand, ground improvement using cement grouting can reduce settlement. However, with the increase in cement grouting slabs, the settlement slightly increases linearly. The increase in cement grouting slab thickness causes an increase in the weight of the slab. Nonetheless, the increase in thickness of lightweight PU foam has caused more water to be displaced in saturated soil equal to the buoyant force that will act in the upward direction; therefore, overburden load that is acting in a downward direction causes settlement of the soft ground to be overcome.

#### 2.7.3. Finite Element Models

**Figure 10:**
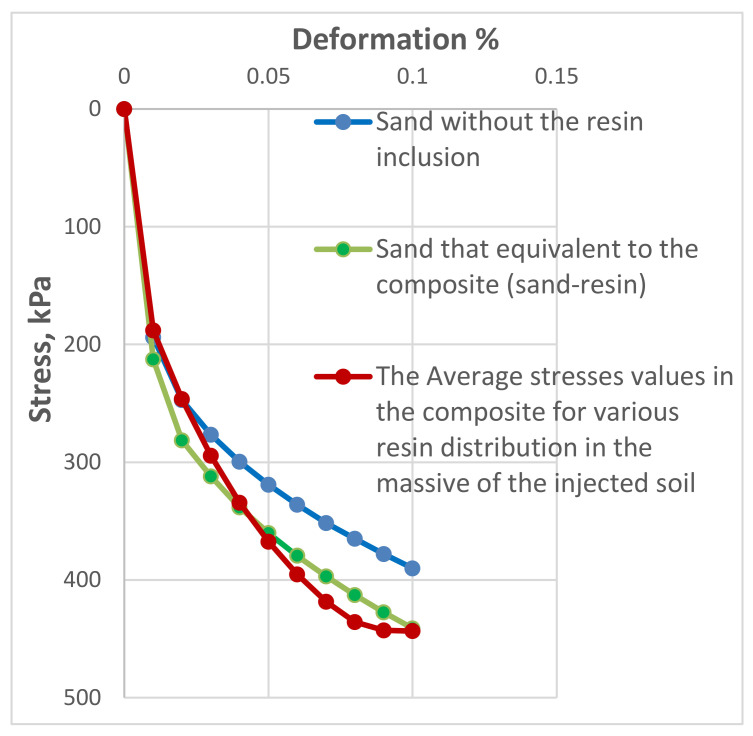
The generated stresses in the soil massive without the resin inclusion in a homogenous environment equivalent to the soil with the resin inclusion [68].

Further, Sabri M.M. [68] has developed a generalized calculation method that predicts the bearing capacity and the settlement of the modified soil, which is equivalent to the composite soil-resin after the injection process using finite element analysis, based on the obtained field and laboratory investigations data, as shown in Figure 10. The average configuration of the resin distribution in the soil massive, after the injection process, has been revealed and considered in the calculation model (Figure 10). The developed model is substantiated by a series of field plate load tests. It was shown that the calculation results reflect the behavior of the injected soil with sufficient accuracy to be applied in field applications.

### 2.8. Durability and Environmental Aspects

There are almost no published studies on the durability of PU foam, which consists of two components that have been used as an injected expandable material. However, various studies on the degradation of polyurethane foam have shown that the material is durable under various weather conditions, minerals, and sea water.

Based on an extensive experimental study that has been conducted by Peter [111] on the accelerated wet aging of two polyurethane polymers for marine applications, lifetimes by 20 years, before a 20% permanent drop in failure stress, are predicted for structures at 50 °C. Detection of hydrolysis on the surface of sea aged samples by FTIR analysis and extraction of plasticizer revealed by weight changes suggest that at least two of the failure mechanisms induced by elevated temperature laboratory ageing represent degradation that might be encountered in service. The FTIR analysis and the accelerated test results indicate that hydrolysis can occur. Nevertheless, an estimation, based on a linear Arrhenius extrapolation, indicates that the timescale for 50% property loss at sea temperatures is more than 100 years. Sea aging results have shown that the polyurethane polymer retains 100% of its initial tensile properties after five years of immersion. The very slow degradation results have shown that the polyurethanes are suitable for long-term underwater applications. Mechanical, rather than physic-chemical, damage is likely to determine their effective serviceability lifetime. In 2002, Rutkowska et al. [112] investigated the degradation of polyester- and polyether-based PUs in seawater for 12 months. It was found that polyurethanes have different degradability in sea water, and this is due to their different network. The polyurethane foam is very resistant to seawater degradation, while polyesters are very prone to degradation under the same conditions. According to Campion et al. [113], UV and ozone attacks lead to different modes of elastomers degradation used in the offshore industry, including mechanical degradation, chemical changes, and cracking.

Howard [114] investigated the degradation of the isocyanate part of polyurethane foams. It was found that degradation does not occur except in some instances. Different studies on the degradation of PUs to fungal attacks [100,101,102] revealed that polyester PU is more vulnerable to fungal and microbial attack than polyether PU. On the other hand, polyether PU is moderately resistant to fungal and microbial attacks. The polyether PU can resist microbial attack when used in regular contact with soil in severe weather environments. Flexible polyester PU can be at risk of attack and damage from fungi and bacteria. According to Huntsman [115], The load-bearing capabilities of polyester PU are decreased, as the enzymes present in microorganisms can split ester bonds.

Cosgrove [116] investigated the involvement of soil fungal communities in the biodegradation of polyester polyurethane. It was revealed that PU is highly susceptible to degradation in different soils, losing up to 95% of its tensile strength. Therefore, different fungi are associated with PU degradation in different soils, but the physical process is independent of soil type. In another study, four species of fungi, Curvularia senegalensis, Fusarium solani, Aureobasidium pullulans, and Cladosporium sp. were isolated based on their ability to utilize a colloidal polyester PU as the sole carbon and energy source

Crabbe [117] studied four isolated soil fungi species (Cladosporium sp. Fusarium solani, Aureobasidium pullulans, and Curvularia senegalensis) on the degradation of ester-based polyurethane. It was found that they lead to degrading ester-based polyurethane in a polyurethane-agar clearing assay. Kay et al. [118] investigated the effect of various bacterial isolates to degrade polyester, by the results of which the author stated that bacteria might play a significant role in the biodegradation and biodeterioration of polyester polyurethane. Further study of Kay et al. [119] was the physical and chemical changes in degraded polyester. A significant reduction in tensile strength and elongation was noticed after three days of incubation for polyurethanes taken from Corynebacterium sp. When exposed to infrared spectrophotometer analysis, it was revealed that the ester segment of the polyurethane is supposed to be the weakest under attack.

### 2.9. Factors Affecting the Selection of the Ground Improvement Technique for Existing Foundations

According to the work and recommendations of many authors [65,120,121,122,123,124,125] and others, various factors affect the selection of the optimum ground modification method in situ. These factors can be sorted as:

- The functions and the degree of improvement required. Different techniques exist globally and provide different functions to solve various foundations problems. Consequently, the required functions and degree of the improvement depend on several aspects, particularly for each project, and are determined by different laboratory and in-situ geotechnical investigations alongside the physical and numerical models obtained. Thereby, selecting the appropriate technology is associated directly with the functions and the degree of improvement required.
- Technical and geotechnical constraints. Such as the type, conditions, and the geological structure of the soil beneath the existing foundations. In general, selecting the appropriate technique depends substantially on the type of the treated soil, the mechanism of the technique used, the materials used for the stabilization, and other aspects. Moreover, the effective depth and extent required to be treated and the groundwater level play essential roles in selecting the efficient technique. Several stabilization methods are limited in complex geotechnical tasks where the depth of the required improvement is extended. Not all existing techniques work efficiently below the groundwater level, and in some cases, the water should be pumped to apply a particular method. The operational load of the overlying buildings and structures is another factor affecting the selection of the appropriate method since though the stresses generated in the soil layers due to the injected solutions should be transferred reliably, ensuring that the additional pressures do not lead to displacing the soil laterally, causing future adverse effects. Furthermore, over lifting risk is one of the most significant factors in selecting the appropriate compensation technique.
- Projects constraints. Such as the available cost, equipment, and materials required for the ground modification of a particular project. Besides, the specifications differ from one country to another, the scope of the constructions, and the space constraints. Different effective techniques are not applicable for small-scale constructions, due to site inaccessibility and insufficient space for construction equipment to operate safely, overhead clearance, and adjacent structures and utilities. These aspects should be considered when choosing the appropriate methods, especially in small-scale projects where the extensive labor and huge equipment might be costly and not valuable compared to the scale of the projects.
- Time constraints: The speed of the improvement technique method plays an essential role in selecting the ground improvement technology in any geotechnical project, especially in vital constructions such as airports, roads, and other infrastructures where it is necessary to choose fast techniques for soil stabilization and remediation process. The closure of such vital infrastructures for maintenance might be very costly and detrimental to these service projects and reflect negatively on the performance of other major or minor infrastructures because of the transferred momentum generated by the closure of major infrastructures.
- The possibility of adverse effects to the foundations and overlying structures. The technique used should be non-destructive to the soil, the overlying structures, and the neighborhood’s foundations. The chemical-based methods might lead to adverse effects due to the chemical reactions with the soil compositions of the minerals contained in the soil massive, leading to various problems such as the expansive soil and other problems, affecting the status of the foundations and the overlying buildings and structures. Thus, assessing the soil’s organic content is necessary to ensure the selection of the proper technique. Moreover, methods that require heavy equipment might not be an optimum solution in the residential areas where this equipment might affect the adjacent foundation due to its vibration besides the noise caused disturbing the neighborhood residents.
- The effect of the technique on the ecology. Some chemical-based methods are restricted due to their adverse effect on the environment, including the contamination of the groundwater level due to the toxicity of the chemical additives. Additionally, the seepage damage due to high pressure, applied by the technique used, is another restriction when selecting the effective method for solving geotechnical tasks. These factors should be considered when selecting the appropriate method for foundations remediation and reinforcement of the foundation’s soil.
- Other factors. Such as the durability and the quality of the injected material. Several factors affect the injected grouts, reducing their long-term performance in the soil massive, such as the groundwater level, soil mineral, and microbial contents, shrinking-swelling of the grout, due to the variation of the temperature, and others. These factors affect the quality and durability of the selected material and the technique’s efficiency, leading to prefer a technique among other techniques for solving particular foundations problems.

### 2.10. The Advantages of the Soil Injection Technology Using an Expanding Resin and the Limitation of Use

There is limited documented research on the use of polyurethane injections for soil improvement. Some suggestions of possible applications of this method, such as use with peat, are not currently backed up with success in the field or lab. Therefore, using polyurethane in such applications may carry more risk than other types of well-documented ground improvement techniques.

Despite the cost of the material, this technology is supposed to be one of the most efficient techniques for rapid settlement compensation with strengthening the soil beneath foundations.

Lists of the advantages and limitations of polyurethane injection for practical application use are summarized in Table 2.

## 3. Discussion

Based on the comparative analysis of the conducted experiments and finite element analysis in this field, this technology provides a superior solution for soil strengthening and settlement compensation. The primary gained results in this field could be concluded as:

### 3.1. Foundations Lifting Results

The effectiveness of the technology, for fast leveling and compensation of the differential settlement of foundations of buildings and structures, has been practically confirmed in different soil types.

- Lifting to 20 mm of differential settlement foundations has been recorded. However, lifting to more values within the range of settlements requiring lifting to a value of which the load-bearing element is a composite (soil and resin) is possible as long as the maximum load of buildings does not exceed 10,000 kPa
- The injection is carried out in shots; thus, the over-lifting risks, due to the hydrofracturing process, are minimized.
- The optimum and most economical monitoring method is a laser level with high accuracy (0.01 mm) during the lifting process.

### 3.2. The Soil Modified Properties

Various studies have been conducted to determine the properties of the modified soils after the injection process. After analyses of the modified physic-mechanical properties of different soil types, the following can be concluded:

- Indicators of the conditional dynamic resistance of the soil are increased after its injection by the expandable resin. The average increase in non-cohesive soil dynamic resistance is up to 81%.
- The average bearing capacity of resin-strengthened soil increases by 67–400%, and the calculated modulus of deformation of the resin-strengthened soil increases by 55–203%. The variation in the soil-resin composite’s final characteristics is justified by the variation in the treated depth, soil type, and other factors.
- Although, the deformation modulus of the composite soil-resin is depth-dependent. Nevertheless, the main advantage of this technology is the possibility of settling sediment, that is, using it as a “liquid jack.” In light of this consideration, settlements are excluded due to the lifting of the foundations by the presence of the resin, and not due to the changes in the deformation modulus.
- The effective distances interval is 1–1.5 m, within which a considerable increase in the soil properties is noticed.
- A 68% reduction in the clay peak swelling pressure has been recorded for soil with as little as 1% macro voids in its total volume.
- The liquefaction potential of the sand is remarkably decreased after the injection of the resin. The evaluation of the dynamic properties of polyurethane resin indicates that this material can be considered a potential alternative for reducing seismic earth pressures. Dilatometer test showed an increase in horizontal stress index KD from 50 to 150% after the resin injection. Shear waves velocity increased around 50%, the relative density increased by 30 %, and the Modulus of Subgrade Reaction (k) increased around 50 to 90% at different injection locations.
- The soil’s shear resistance is noticeably increased up to 257% after the injection of the resin. However, this increase depends on the final formed density of the injected resin.
- The moisture content, the particle size distribution, and the specific gravity are improved due to the resin inclusion, accompanied by an impressive reduction in the existing voids and swelling index after the injection.
- The soil cohesion increases by 187–260% in non-cohesive soils due to the resin inclusion into the soil massive. However, the modified properties of cohesive soils are not yet determined.
- The injected resin is non-toxic, and there is no possibility for any chemical reaction with the soil’s expansive minerals.

### 3.3. The Resin Propagation

During several years of research, a misleading determination of the correct configurations of resin propagation in the soil massive. The analysis of the resin propagation in the soil massive has shown the following:

- Although some conducted research stated that the flow and propagation of the PU expandable foam are subjected to the cavity expansion theory when injected into the soil massive, this theory is considered if the resin is injected at low injection pressure. In this case, the resin expands not along the fracture but in the form of a ball or cylinder. Consequently, it leads to high resin consumption and the inability to achieve a uniform distribution for the foundations lifting and turns out to be ineffective.
- In the practical applications, the injected resin diffuses in both non-cohesive and cohesive soils, forming identical continuous walls of foam plates along the whole injected depth, surrounding the injected soil from all sides, connecting at the edges of the resin within a distance interval around 30–50 cm, and the average thickness of the resin is 0.8–25 mm, approximately, as measured. Figure 2, Figure 3 and Figure 4 show different sections of the resin propagation in the massive of various investigated soils.
- The peculiarity of the two-component injection technology is the rapid solidifying process of the resin. During the hardening process, the pores of the sand are calmed, and the injection material spreads in the form of hydraulic fracturing made by the injection pressure. Thus, the resin’s diffusion through the pores, due to the filtration of the soil, practically does not occur.

### 3.4. The Resin Properties

After the resin is injected, the resin and the soil form a composite material where the resin acts as a bearing element. Thus, the mechanical properties of the formed resin, based on its volumetric expansion ratio, play an essential role in the properties of the formed composite. Analysis of the mechanical properties of the resin has shown the following:

- The relationship between density and ultimate strength (σ = 48.3 ρ2 + 2.54ρ) and the relationship between density and modulus of elasticity (*E* = 325.17 ρ − 10.99) of the expandable resin within the density range (from 0.053 up to 0.354 gm/cm^3^) and the volumetric expansion coefficients of the resin (from 3 to 15), respectively.
- The average density of the resin formed in the non-cohesive soils is 0.184 gm/cm^3^ at an injection pressure of 100 bar and a temperature of 15 °C. There is no comparative registered data in the cohesive soils.
- The resin can be formed in various densities, allowing a high spectrum of the mechanical properties in the soil massive up to (*E* = 8–73 MPa, σ = 0.5–4.6 MPa), respectively, at expanding ratios ranges (3–15 times). Consequently, this resin is considered a high-strength injected material compared to various injection materials used for soil stabilization, considering the rapid lifting and strengthening processes.

### 3.5. The Optimum Resin Consumption

The optimum resin consumption in the sandy soils is 10 L per 1 m^3^, which is equal to 1% of the total soil volume. Changing the consumption rate leads to changes in the resin density in a homogeneous injection environment. Consequently, the resin properties are changed, affecting the final modified soil-resin composite properties. However, a lack of estimation of the resin consumption in other soil types is still existing.

### 3.6. Finite Element Models

A finite element model for calculating the bearing capacity and assessing the settlement of the foundations after the injection process in the hydrofracturing mode has been developed. In turn, it improves the application of this technology and increases its efficiency and operational reliability. The reliability of the proposed model was confirmed by comparing the numerical results with the results of full-scale plate load tests. The comparison results have shown that the developed model reflects the actual behavior of the composite with sufficient accuracy. The model considers the average resin diffusion under a homogenous injection environment. It considers the density and the form of the expanded resin in the soil massive, which allows for estimating the actual consumption of the resin propagation required to gain specified strengthening.

## 4. Future Work

Most research on this technology aims to investigate the effect of the expandable polyurethane resin on soil stabilization and foundation restoration. As a result, the effectiveness of resin for the settlement compensation and strengthening the foundation’s soil was proven. However, further research may be directed for the future improvement of the application of this technology.

1. A calculation method of the settlement and the bearing capacity of the modified composite (soil-resin) properties has been developed and validated [72]. However, generalization and validation of this method for other soil types are still required.
2. The solidified resin’s density formed in the soil’s massive after the injection process is the most significant factor that affects its mechanical properties, thereby identifying the degree of the soil strengthening required after the treatment. The resin’s density varies due to its expansion properties, and the volumetric amount of the resin used plays a role in the final density formed. Therefore, a developed method that accurately predicts the resin’s density after the injection process is necessary for the practical applications
3. The deformation modulus of the composite (soil-resin) is varied along with the depth of the soil. Further investigations of the changes patterns in the composite’s deformation modulus at different depths are required.
4. To investigate the effect of the component compositions ratios variation on the modified properties of the composite is necessary.
5. Since it was established, this technology has been used for shallow foundations due to various limitations. The development of equipment and procedure for using this technology to remediate and strengthen deep foundations (below 10 m) is a significant task for future technology applications.
6. The variation of the surcharge load applied to the soil significantly influences the resin’s physic mechanical properties and consumption. Investigating this effect using large-scale field and laboratory experiments can improve the practical applications of this technology and reduce resin consumption.
7. The optimum resin consumption in the sand has been determined. However, further research on the optimum resin’s volumetric consumption rate, for strengthening the cohesive soils, is still obligatory for generalizing the results.

## 5. Conclusions

1. Various techniques are used for soil strengthening and compensation of the foundation’s settlements of the existing buildings and structures. Each technique has an application field based on its functions and the accessibility of use under different project conditions. Some of the significant limitations for most of the traditional techniques are the type of treated soil, the project constraints, and the time constraints fundamentally in vital infrastructures which require a rapid reconstruction process as the traditional materials composition used in most of the existing techniques have a long curing process and require tremendous preparation prior the treatment.
2. The soil injection, using an expandable polyurethane resin, provides a vast solution for foundation lifting and settlement compensation, strengthening the soil beneath. However, very few experimental investigations regarding this technology have been conducted worldwide.
3. The effectiveness of the reviewed technology for fast foundations, lifting and compensation of the differential settlement of buildings and structures, has been practically confirmed in different soil types (Section 2.7).
4. Analyses of the modified properties of different soil types (Section 2.7) have revealed that the injection of the soil by an expandable resin leads to significant improvements of the physic-mechanical and hydraulic properties of the treated soil. Besides that, the soil-resin acts as a composite material where the resin serves as a bearing element.
5. The analysis of the resin propagation in the soil massive has shown that the resin propagates in the soil massive through the fractures made by the hydrofracturing mode rather than the cavity expansion (Section 2.5).
6. The optimum resin consumption in the sandy soils is 10 L per 1 m^3^, equal to 1% of the total soil volume (Section 2.6). Changing the consumption rate leads to changes in the resin density in a homogeneous injection environment. Consequently, the resin properties are changed, affecting the final modified soil-resin composite properties.
7. A practically confirmed method for calculating the bearing capacity and assessing the settlement of the foundations after the injection process in the hydrofracturing mode has been developed (Section 2.8). The calculation method considers the average resin diffusion under a homogenous injection environment. In turn, it improves the application of this technology and increases its efficiency and operational reliability.
8. The relationships between mechanical properties of the expandable resin and its density, based on its volumetric expansion properties varied out by the amount of the injected resin have been established (Section 2.7). Further, the density of the resin, under a certain injection environment, has been determined and fixed by 0.184 gm/cm^3^ (Section 2.4).
9. This technology has many advantages, such as the rapid and highly controlled foundations remediation process, due to the rapid curing of the injectable resin, and the ability to use in almost all soil types. The proposed resin does not contain any particles that the soil porosity can restrict. Moreover, the ease to use, the high mobility, the lightweight of the injectable expandable resin and its non-toxic features, and the independence of the resin physic-mechanical properties, from the groundwater level, allows the application of this technology under different geotechnical constraints and projects conditions.

## Figures and Tables

**Table 1:** The resin consumption at each injection point was recorded in-situ during the injection process [68].

The Injection Point	The Consumption Required for the Strengthening, (Liters)	The Consumption Required for Lifting, (Liters)	The Looses (Liters)
Point 1	22	9	3
Point 2	19	7	2
Point 3	37	17	7
Point 4	17	13	3
Point 5	28	11	5
Total	123	57	20

**Table 2:** The advantages and limitations of the technology in practical applications.

Advantages [64,66,68,70,71,76,78,83,93,95,97,103,104,105,106,110,126,127,128,129].	Limitations [64,65,67,70,72,74,83,128].
The technology can treat the soil within specified spots or the entire geotechnical site.	A limited injection depth. Up to 10 m in the soil’s depth, limiting the implementation of the technology to be used certainly for shallow foundations.
2. The ability to rapidly lift the settled building and structures and strengthen the treated soil zones provides a high functionality under various geotechnical conditions.	2. The density of the resin in the soil massive after the injection process is non-predictable without having laboratory tests or reference injection environment data for the modeling process. Thus, careful design issues should be considered regarding soil treatment using this technology to gain effective results.
3. The resin has lightweight relative to the soil; thus, it causes no further settlement in the soil layers beneath the treated zones.	3. The propagation of the resin is a random phenomenon in different soil types. Thus, the forms and configurations of the resin distribution should be investigated in different soil types as it affects the final strength results of the composite soil resin.
4. The results are gained immediately due to the fast chemical reactions of the resin components; thus no time constraints when this technology is implemented.	4. The documentation and published detachers available are rare compared to other techniques.
5. The high mobility and small equipment size allow the use of this technology regardless of the scale and space constraints of the constructions; thus, project constraints are minimized when this technology is implemented.	5. Susceptible to UV light and microbial degradation
6. It could be used for different soil types regardless of the soil’s porosity index due to the liquidity and the fluidity of the resin’s component, which contains no particles that the soil porosity might limit.	6. Relatively high material cost.
7. The resin provides a high spectrum of mechanical properties depending on its final density controlled by the injected amount; therefore, it allows gaining the desired results within the composite frame properties.	
8. The injection process is controlled by the injection “in shots” successively, preventing over-lifting risks and fine-tuning the foundation to the desired elevations.	
9. The improvement of the soil properties is ranged between 200–400% of its initial properties in different soil types; thus, the technology is effective in terms of its use for soil reinforcement.	
10. The technology provides non-destructive settlement compensation and soil reinforcement during the reconstruction process.	
11. Ease to design and inject compared to other techniques.	
12. The fast resin curing allows remediating vital infrastructures such as airports, highways, and railways due to the nature of its operations, as any closing of these facilities lead to severe problems.	
13. The lifting to the desired results is a delicate and precise operation through a successive injection “in shots.” The uplifting process is carefully monitored by laser level. The results are gained immediately, leading to a considerable saving in both time and cost.	
